# Metrics, Dose, and Dose Concept: The Need for a Proper Dose Concept in the Risk Assessment of Nanoparticles

**DOI:** 10.3390/ijerph110404026

**Published:** 2014-04-14

**Authors:** Myrtill Simkó, Dietmar Nosske, Wolfgang G. Kreyling

**Affiliations:** 1 Institute of Technology Assessment, Austrian Academy of Sciences, Strohgasse 45, Vienna 1030, Austria; 2 Environmental Resources and Technologies, Health and Environment Department, Austrian Institute of Technology GmbH, Konrad-Lorenz-Straße 24, Tulln 3430, Austria; 3 Department Radiation Protection and Health, Federal Office for Radiation Protection, Ingolstädter Landstr. 1, Oberschleißheim 85764, Germany; E-Mail: dnosske@bfs.de; 4 Institute of Epidemiology II, Helmholtz Zentrum München, German Research Center for Environmental Health (GmbH) Ingolstädter Landstr. 1, Neuherberg/Munich 85764, Germany; E-Mail: kreyling@helmholtz-muenchen.de

**Keywords:** nanotoxicology, radiation biology, deposited nanoparticle dose, dose rate, equivalent dose, effective dose, nanoparticle surface area

## Abstract

In order to calculate the dose for nanoparticles (NP), (i) relevant information about the dose metrics and (ii) a proper dose concept are crucial. Since the appropriate metrics for NP toxicity are yet to be elaborated, a general dose calculation model for nanomaterials is not available. Here we propose how to develop a dose assessment model for NP in analogy to the radiation protection dose calculation, introducing the so-called “deposited and the equivalent dose”. As a dose metric we propose the total deposited NP surface area (SA), which has been shown frequently to determine toxicological responses e.g. of lung tissue. The deposited NP dose is proportional to the total surface area of deposited NP per tissue mass, and takes into account primary and agglomerated NP. By using several weighting factors the equivalent dose additionally takes into account various physico-chemical properties of the NP which are influencing the biological responses. These weighting factors consider the specific surface area, the surface textures, the zeta-potential as a measure for surface charge, the particle morphology such as the shape and the length-to-diameter ratio (aspect ratio), the band gap energy levels of metal and metal oxide NP, and the particle dissolution rate. Furthermore, we discuss how these weighting factors influence the equivalent dose of the deposited NP.

## 1. Introduction

It has been estimated that the number, variety, and assortment of applications of engineered nanoparticles (NPs) will increase rapidly over the next few years [1]. Due to their intrinsic properties, NPs are commonly used in a variety of areas, not only in electronics, photovoltaics, catalysis, engineering, cosmetics but also in medicine and pharmacy. Studies have shown evident toxicity of some NPs to living systems but because of the lack of knowledge of all the relevant toxicological mechanisms, a comprehensive risk assessment cannot be performed yet. There is a need for using novel and fast techniques such as high-throughput methods to test the potential toxicity of these materials. However, at this time tests are very expensive and time consuming [1]. Regulatory agencies, however, must be able to act also in the absence of complete toxicological data sets of NP effects. Therefore, appropriate NP dose model(s) have to be identified to facilitate dose assessment. 

There are different drivers of toxicological studies: the intrinsic chemical, the material morphology, and the radiation driven toxicity, which all use clearly defined metrics. It has been proposed that in nanotoxicology the total surface area of the NP and the NP surface reactivity are relevant dose metrics for toxicity. It is known that nanomaterials can induce significant toxic effects due to their physico-chemical properties. These include the surface of the material which can be involved in catalytic and oxidative reactions that may subsequently induce cytotoxicity [2], but there are also other properties, such as the NP shape, charge, *etc.* which influence the biological effects of NPs. Frequently, the observed toxicity was reported to be greater for nanoscale materials than that of bulk forms of the same material, which may be caused e.g., by their much larger specific surface area (SSA), *i.e.*, surface area-to-volume ratio. Here we suggest a NP-dose model which considers the total deposited NP surface area (SA) per tissue mass. 

In the human body, three main portals of entry for nanomaterials are usually considered: the respiratory, the digestive, and the cutaneous (skin). The fourth portal of entry, direct NP injections into blood circulation in nanomedicinal applications, is not considered in the present paper. It has been shown in inhalation studies [3,4], that the majority (>90%) of deposited NPs in the alveolar region of the lungs is retained and gradually translocated into the circulation and subsequently accumulated in other organs/tissues of the body. In fact, this pathway of exposure has been studied more intensely than the other pathways. Regarding the uptake of NPs through the gastrointestinal tract, the number of available studies is relatively modest although the oral uptake of NPs, e.g. as supplements in food has increased steadily over the last decades. However, Bergin and Witzmann summarized in a recent review, that it is unlikely to have any toxic effects at typical levels of exposure via ingestion of NPs [5]. Current evidence for the uptake of NPs through the skin is very low and, therefore, we do not include this pathway into our considerations. Thus, here we only focus on the deposited dose of NPs after inhalation.

As already mentioned, the toxicity of nanomaterials is dependent on their physico-chemical properties. There are mathematical tools available, such as Quantitative Structure Activity Relationship (QSAR) models, which attempt to predict the potential NP toxicity depending on the physico-chemical characteristics of the material. QSAR also maximizes the understanding from existing experimental data to enable predictions for additional compounds that have not been tested [6]. Burello and co-workers [7,8,9,10,11,12], but also Nel and his group [13,14], discussed several applications of QSAR for nanomaterials. However, the application of nano-QSAR is still limited because of the complexity and potential “unknowns” of the nanomaterials (relating to properties, interferences with environmental and biological matrices, dynamic changes *etc.*) [10]. Puzyn *et al.* [15] have shown by using the QSAR concept and *Escherichia coli* as the test model, that QSAR is a partly applicable model for predictive toxicity of a small set of nanosized metal oxides. Gajewicz *et al.* [16] reviewed the state of the art of the different predictive computational approaches and recommended a cooperative approach between computational scientists and toxicologists with a view to develop predictive toxicology for NPs in order to design safe nanoproducts. A recent review [17] listing major knowledge gaps suggested an increased use of QSAR methods to predict NP toxicity. However, it is not clear yet, which properties of the NP are relevant for computational approaches, and there is still ongoing discussion about the appropriate biological endpoint(s) for NP effects. 

Many studies have investigated a variety of endpoints in different *in vivo* and *in vitro* systems (see e.g. [18,19]). However, oxidative stress is recognized as one of the central mechanisms by which NPs might induce toxicity [3,20,21] (even though other *in vitro* and *in vivo* endpoints of toxicity may deal with different toxic effects). The formation and release of reactive oxygen species (ROS) is closely connected to the immune defense system whereby high levels of ROS can lead to a number of damaging pathological consequences such as lipid peroxidation, protein damage, deactivation of enzymatic activities, and DNA modification and general pro-inflammatory processes. In the normal cellular biochemistry there is a balance between free radical formation and the action of an antioxidative system. Accordingly, oxidative stress caused by NPs plays a decisive role in cytotoxicity and inflammation, eventually leading to the onset of pathophysiological alterations and pathogenesis. Consequently we focus on this endpoint in the present model.

The goal of this paper is to present a concept for dose assessment of nanoparticles using the total surface area of NP as a dose metric. This concept is influenced to a large degree by the dose concept developed within the field of radiation protection over the last six decades, and it has also the potential to complement nano-QSAR models. Accordingly, the presented model takes into account:

(1) *The deposited dose*, which is the total deposited NP surface area (SA) per tissue mass or volume (m^2^/kg, or m^2^/m^3^ or m^−1^). This SA of the NP has the potential to induce biological effects. Moreover, we consider agglomerated NPs as well.
(2) *The equivalent dose*, whereby the deposited NP dose is weighted by factors quantifying the effects of several other physico-chemical properties of the NPs, such as the specific surface area, surface texture, electron band gap interval at the NP surface, surface charge (zeta-potential), NP morphology (shape, surface roughness, length-to-width ratio (aspect ratio)), and the dissolution rate.

## 2. Dose Assessment for Ionizing Radiation

### 2.1. Exposure to Ionizing Radiation

Humans can be exposed to ionizing radiation by external irradiation from the environment (atmosphere, soil, space,), e.g., from contaminated air or from radioactivity deposited on the ground (external exposure), or by internal irradiation from radionuclides taken up into the body, for example by inhalation, ingestion or injection (internal exposure).

For external exposure, only penetrating radiation (gamma radiation, and to a lesser extent high energy beta radiation) is relevant for dosimetry because non-penetrating radiation such as alpha particles do not reach radiation sensitive cells in the body. However, this non-penetrating radiation is very important for dosimetry in the case of internal exposure, especially for those organs where the radionuclide accumulates.

While external exposure can be stopped immediately by shielding or by moving to non-contaminated places, internal exposure cannot be stopped until the incorporated radionuclides have been excreted or have been eliminated by decay. In the context of this paper nanoparticles taken up into the body may be compared to internal radiation exposure.

### 2.2. Absorbed Dose of Ionizing Radiation

The fundamental dose quantity in radiation protection is the absorbed dose, *D*:

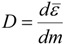
(1)
with the mean energy *dε* imparted to matter of mass *dm* with the unit J·kg^−1^ or the special unit gray (Gy).

In radiation protection, the organ or tissue absorbed dose, *D_T_*, which is the mean dose to a target organ or tissue *T*, is often used. For internal exposure *D_T_* is expressed by the committed dose to T for a defined time period *τ* (taken to be 50 years for adults). Then:

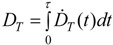
(2)
where *Ḋ_T_*(*t*) is the mean absorbed dose rate in *T* at time *t* (Table 1).

For the calculation of organ absorbed doses for internal exposure, specific information about physical and chemical properties of the radionuclide is relevant. These properties include if the radionuclide is contained in solid, liquid or gaseous chemical compounds, the particle size in case of radionuclides contained in particles, the type and energy of radiation, and the retention time which may be characterized by the effective half-life of the radionuclide. In case of inhalation, the “Human respiratory tract model for radiological protection” of the ICRP [22], and the “Human alimentary tract model for radiological protection” [23] for gastrointestinal exposure are presently used as biokinetic models. With these models—together with systemic and excretion models—the numbers of nuclear transformations in source regions, body regions where the radionuclide accumulates, can be estimated. This information is in turn an input for dosimetric models to calculate absorbed tissue doses. 

**Table 1:** Dose quantities for radiation and in analogy for nanotoxicology.

Dose quantities	Radiation	NP
**Absorbed/deposited dose (*D*): **	The absorbed dose (*D*) is the mean energy *dε* imparted to matter of mass *dm* by ionising radiation, J·kg^−1^ or Gy: 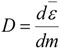	The deposited dose of NP (*D_NP_*) is the total deposited *surface area* (SA) of NP per mass of living matter *dm*, m^2^/kg: 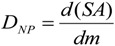
**Dose rate:**	The mean absorbed radiation dose rate in *T* at time *t*: *Ḋ_T_*(*t*)	The mean absorbed NP dose rate in *T* at time *t*: *Ḋ_NP,T_*(*t*)
**Committed tissue dose:**	The quantity of radiation absorbed per unit time: 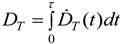	The quantity of absorbed NP dose (uptake) per unit time: 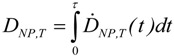
**Equivalent dose:**	The mean absorbed dose from radiation R in a tissue or organ *T*, multiplied by the radiation weighting factor w_R_, J·kg^−1^ or Sv: 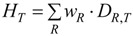	Absorbed dose weighted by NP property(material) dependent reactivity weighting factor(s) w*_PC_*_,*i*_ (*i* = 1, 2, ….) for different physico-chemical properties or functional “behaviors” of NP: 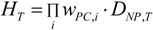
**Effective dose:**	The tissue-weighted sum of the equivalent doses in all specified tissues and organs of the body, J·kg^−1^ or Sv: 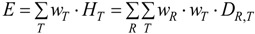	The tissue-weighted sum of the equivalent doses in all specified tissues and organs of the body: 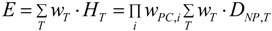

### 2.3. Equivalent Dose of Ionizing Radiation

The likelihood of radiation effects depends on the absorbed dose and on the type and energy of the radiation. For stochastic effects caused by different types of radiation this is expressed as equivalent dose (H*_T_*), whereby the quality of radiation is adjusted by dimensionless radiation weighting factors [24,25]. These factors were determined by comparing the induced biological damage of the radiation to that with an equal dose of gamma or X-rays. X-rays and gamma rays have a weighting factor of 1 (1 Gy = 1 Sv), whereas e.g., for the same absorbed dose, alpha particles are considered to be 20 times biologically more potent for stochastic effects [24,25]:

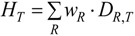
(3)
where *D_R,T_* is the absorbed dose from radiation *R* in a tissue or organ *T*, and *w_R_* the radiation weighting factor.

### 2.4. Effective Dose of Ionizing Radiation

There are also different likelihoods for the occurrence of radiation effects/damages in different organs and tissues due to different tissue sensitivities [26]. For effective dose calculation, individual organ dose values are multiplied by the respective tissue weighting factor. The sum of all organ weighting factors is 1. The present tissue weighting factors are 0.12 each for the red bone-marrow, colon, lung, stomach, breast, and the specifically defined so called remainder tissues [25]. Furthermore, 0.08 for the gonads and 0.04 each for bladder, esophagus, liver, thyroid and finally 0.01 each for bone surface, brain, salivary glands, and skin [25]:

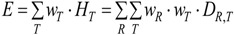
(4)
with tissue weighting factors *w_T_*.

## 3. Dose Assessment for NPs

### 3.1. Exposure to NPs

Exposure assessment is a major component of dose and thus risk assessment. In case of NP exposure, only internal exposure is relevant, since only NPs that have been taken up into the body can cause a dose and toxicity. The uptake of NPs can be dermal, by inhalation or ingestion, but also through intravenous injection for medical applications. Several exposure scenarios can be considered: (i) occupational exposure during NP processing; (ii) exposure to consumers by products containing nanomaterial; (iii) exposure by medical applications and (iv) unintentional exposure to the public, e.g., by accidental pollution during production processes. The majority of studies aiming to quantify NP exposure conditions has been performed in occupational settings, and was focused mainly on inhalation exposure. Exposure outside working places are not yet studied; only few modelling studies that aim to predict environmental and consumer exposure are available [27,28]. 

### 3.2. Dosimetry for NPs

#### 3.2.1. Deposited Dose of NP

As mentioned above, the *absorbed dose* for ionizing radiation estimates the deposited energy per mass of living matter, and the dose rate. In analogy for NP-dose estimation, the deposited dose of NPs (*D_NP_*) is the total deposited *surface area* (SA) of NP per mass of living matter (*m*):

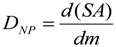
(5)

The *dose rate* *Ḋ_NP_*(*t*) is one more important variable (or entity) which has to be taken into account [29]. Factors affecting the dose rate include the biodegradability of certain NPs. For highly biodegradable NP the time for biological response is relatively short, whereas other NPs may not be degraded within the body. In addition, fractions of NP, depending on their physico-chemical properties, may be retained in the organ where they originally were deposited after intake or they may be excreted, whereas others may translocate into the circulation for biodistribution and subsequent accumulation in secondary organs or for excretion. In analogy to the absorbed dose in radiation dosimetry, the deposited NP dose in a given organ or tissue, *D_NP_*_,*T*_ is adopted. This is the cumulated mean dose to a target organ or tissue *T*. The dose *D_NP_*_,*T*_ is expressed by the committed dose to *T* for a defined time period *τ*. Then:

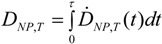
(6)
where *Ḋ_NP,T_*(*t*) is the mean absorbed dose rate in *T* at time *t* (Table 1). 

As for radiation dose assessment, *biokinetic models* for absorbed dose calculations are needed also for NP. In analogy, the “Human respiratory tract model for radiological protection” of the ICRP [22] can be used for the estimation of deposited doses of inhaled NP in the respiratory tract. The multiple-path particle dosimetry (MPPD) model is an advanced modeling software partly based on the ICRP model [22]; it considers exhalation and takes into account the deposition and clearance of aerosols in the respiratory tract. Particles deposited initially in the respiratory tract during inhalation are partly transported to the larynx by mucociliary action out of the lungs and the extrathoracic airways and swallowed into the gastrointestinal tract. ICRP has developed a “Human alimentary tract model for radiological protection” [23] which could be useful for NP dose calculation for the gastrointestinal tract. 

#### 3.2.2. Equivalent Dose of NP and Weighting Factors

In analogy to radiation weighting factors (*w_R_*) for deposited radiation energy, we now propose weighting factors (*w*) which relate to specific quantifiable physico-chemical (PC) properties of the NP. Hence, and in contrast to the *w_R_* in radiation dosimetry, there will be several weighting factors *w_PC,i_* (*i* = 1, 2, ...) for NP which may be constants or functions of NP parameters. 

Accordingly, the equivalent dose *H_NP_*_,*T*_ for each organ or tissue T will be defined similarly to Equation (2). In other words, we will use the term “equivalent dose for NP” considering the “bioactivity” (or a precursor of equivalent dose) of the NP. The equivalent dose is the product of *w_PC,i_* multiplied by the deposited dose. Since the weighting factors have been assigned to characteristics of the NP which all influence the total absorbed dose, here the product of weighting factors has been chosen to enable that each weighting factor separately influences the dose according to the biological impact of the respective NP characteristics. Table 1 shows a comparison between the equations for ionizing radiation and NPs:

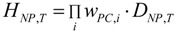
(7)

Introducing this concept of weighting factors *w* for different PC properties of NPs will allow for the estimation of the equivalent dose *H_NP,T_* as a function of the deposited NP surface area and of equivalent dose comparisons between various engineered NPs and their PC properties. We consider that different NP properties are associated with the induction of biological effects. The majority of studies which have investigated biological endpoints in nanotoxicology find clear correlations between the induction of ROS and nitrogen radicals and our dose metric, namely the deposited NP surface area. 

With this dose concept for deposited NPs using the total deposited surface area as the dose metric, a rationale-based and more quantitative ranking of possible effects of NPs in biological tissues will be possible. As PC properties characterized by weighting factors we especially consider: (1) the *specific surface area*, (2) the *surface textures*, (3) the *zeta-potential* as a measure for surface charge and (4) the *particle morphology* such as the shape and the length-to-width ratio (aspect ratio). (5) Furthermore, *the band gap energy levels* of metal and metal oxide NP are considered where oxidative stress is caused by NPs. The oxidative stress is based on the relationship between the cellular redox potential and the band gap energy levels. A further relevant physico-chemical property is (6) *the particle dissolution/dissociation rate*. The proposed *w_PC,i_* can be extended and/or modified and adapted according to the available data and emerging knowledge. In the present model the reactivity of free radicals is considered to be the most relevant biological response for dose estimation. Furthermore, functionalized and/or surface modified NPs may represent “new” NPs with their own surface area and physico-chemical properties.

1. Proposal for the first weighting factor *w_PC_*_,*1*_: The *specific surface area* (SSA), *i.e.*, the ratio of NP surface area to the NP volume, increases with decreasing particle diameter. One single nanoparticle can contain atoms/molecules from a few to several orders of magnitude higher numbers of atoms/molecules. As a result, a certain fraction of the atoms/molecules is located on the particle surface depending on the size, the shape and the physical structure like roughness/smoothness, *etc*. Since it has been shown in many recent papers that the specific surface area is most relevant for the induction of oxidative stress, we suggest the first weighting factor w*_PC,1_* to be related to the fraction of atoms/molecules on the surface of NPs relative to the total number of atoms/molecules. 

For example, for a spherical NP with the diameter d the volume is π/6 d^3^, and the spherical surface area SA is π d^2^; hence, the specific surface area SSA becomes:

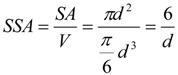
(8)

Then the weighting factor is a function of the inverse NP diameter d:

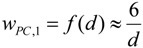
(9)

In the following we show how the fraction of atoms/molecules on the NP surface *N_SA_* per total number of atoms/molecules *N_tot_* within the NP will change with NP size *d_NP_* and the atomic/molecular volumes *V_mol_* of a number of relevant metal and metal oxide NPs. This fraction will be proportional to the weighting factor affecting biological effects:

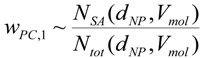
(10)

**Figure 1:**
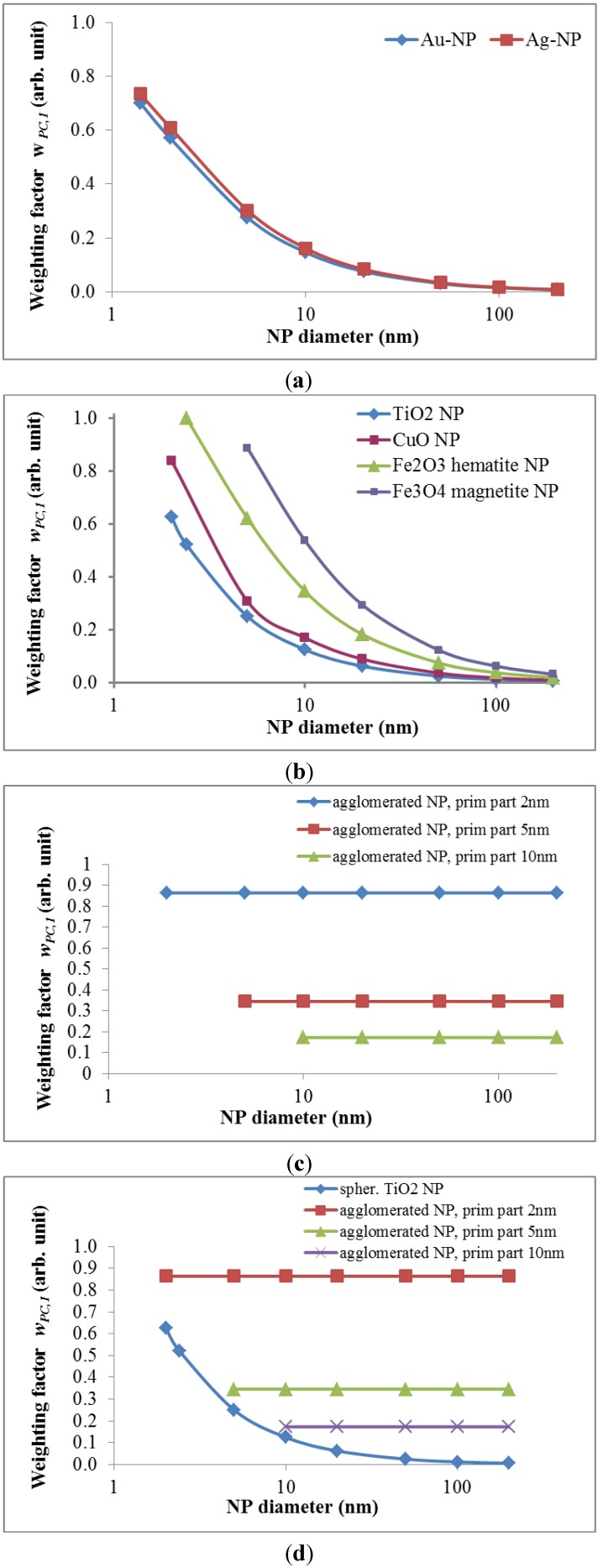
Weighting factor 1 in arbitrary units which is proportional to SSA—*i.e.*, the ratio of atoms/molecules at the NP surface over the total number of atoms/molecules: (**a**) gold (Au) and silver (Ag) NP, (**b**) TiO_2_, CuO, Fe_2_O_3_, and Fe_3_O_4_, (**c**) for agglomerated NPs with 2, 5 and 10 nm primary particle size, (**d**) spherical and agglomerated TiO_2_ with different primary sizes. Panels (c) and (d) show that w_*PC,1*_ is constant for different sizes of agglomerates but changes with the SSA of primary particles.

This ratio is shown for gold (atom diameter 0.29 nm) and silver (atom diameter 0.32 nm) in Figure 1a. Since the gold atoms are smaller than the silver atoms, the fraction of gold atoms on the surface is slightly lower compared to that of the silver atoms.

Similarly, crystallographic data on the molecular dimensions can be used to estimate the number of various metal oxides molecules on the surface as well as within the NP lattice; this is shown for spherically shaped NPs of titanium dioxide, gamma Fe_2_O_3_ hematite, Fe_3_O_4_ magnetite and monoclinic CuO in Figure 1b. Since the tetrahedral structure of the TiO_2_ molecules is smallest, their fraction of atoms on the surface relative to the total number of TiO_2_ molecules in the NP is smaller than those of the other metal oxide molecules; vice versa the surface fraction of Fe_3_O_4_ magnetite is highest since these are the largest of the selected metal oxide molecules.

While this *w_PC_*_,*1*_ is declining rapidly with increasing size of the NP (see Figure 1a,b), *w_PC_*_,*1*_ behaves different for *agglomerates* of primary nano-scaled particles. Agglomerates coagulate, e.g. due to weak forces like the van-der-Waals force or magnetic forces in case of magnetic nanoparticles, such that their contact points are infinitesimally small [30]. As a result the surface area *SA_agg_* of an agglomerate is the sum of the surface areas of all primary particles *SA_pp_* within an agglomerated particle; similarly the volume of the aggregate is the sum of all volumes of the primary particles. If all primary particles are of the same size then they all have the same surface area *SA_pp_* and the same volume *V_pp_* or mass and, hence, the same specific surface area *SSA_pp_*. Applying this to an agglomerated particle of n identical primary particles, the agglomerated particle has the same specific surface area *SSA_pp_* as the primary particles independent of the number of primary particles within an agglomerated particle:

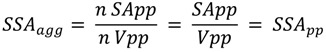
(11)

Hence *w_PC_*_,1_ becomes a constant:

*w_PC_*_,*1*_ = f (*SSA_pp_*) = *c_agg_* for agglomerates of same-sized primary particles (12)

where *c_agg_* is a constant which differs for different sizes of primary particles. 

If the primary particles are not all of the same size but show a size distribution, then their agglomerates may differ in their specific surface area. Yet when considering a large enough number of agglomerates, they will have a specific surface area *SSA_agg_* which is equal to the average of the specific surface area *SSA_pp_* of the primary particles and this is independent of the actual size of the agglomerate [31]. 

Hence, for agglomerates containing a large enough number of primary particles with a mean *SSA_pp_** w_PC_*_,1_ is also a constant *c’_agg_* depending solely on the mean *SSA_pp_* of the primary particles (Figure 1c,d)

*w_PC,1_* = f **(***SSA_pp_***) = *c’_agg_*** for agglomerates (13)

2. Proposal for the second weighting factor *w_PC_*_,*2*_: *w_PC_*_,*2*_ represents an arbitrary weighting factor for the *surface* *texture*. For spherical NPs with smooth surfaces *w_PC_*_,*2*_ is set to unity *w_PC_*_,*2*_ = 1 while *w_PC_*_,*2*_ becomes *w_PC_*_,*2*_ > 1 for irregularly shaped NPs with sharp edges and ridges and a rough surface. This surface roughness relates to the fact that atoms/molecules on the rough surface of NP are less integrated into the NP lattice and may be “unsaturated”, such that they are more prone to electron transfer than the inner atoms/molecules of the NPs leading to oxidative stress reaction in biological systems. In addition, their relative number to the total number increases due to the increasing surface area; hence, free radical production may increase and/or these atoms/molecules may change their stoichiometry and/or they may even leave the NP surface such that the remaining NP surface texture may increase in surface reactivity.

For spherical NPs curvature increases drastically with decreasing size and is proportional to the inverse of the diameter *d* of the NP as discussed above. In analogy of the Young-Laplace equation which relates pressure *p* on the surface of a liquid sphere to surface tension σ and the inverse of the diameter:

*p* = 4 σ/*d* (14)

we can introduce a surface module σ’ which describes the probability of atoms/molecules at the surface to either alter their electron shell or change their molecular composition (which also leads to altered electron shells) or even escape the surface. The probability will be greater than the probability σ_0_‘ for atoms/molecules inside the NP. In the absence of precise data we propose for *w_PC,2_*:

*w_PC,2_*~(σ‘/σ_0_‘)/d with 1 < σ‘/σ_0_‘ < 10 (15)

**Figure 2:**
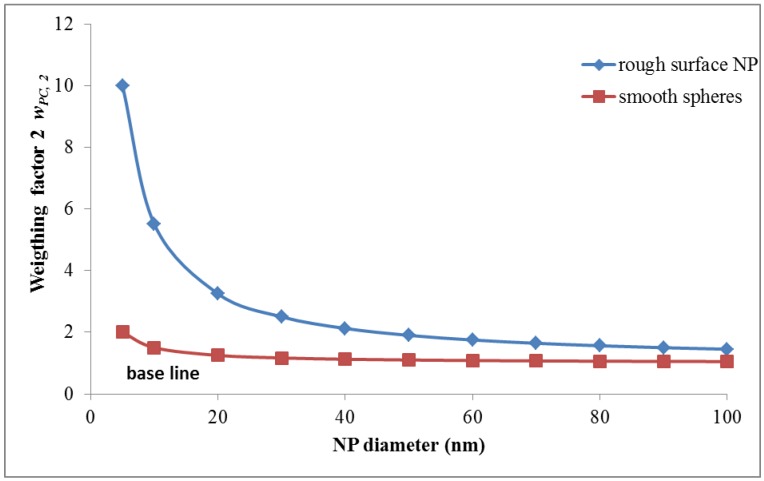
Weighting factor 2 (*w_PC,2_*) is proportional to the surface texture of NPs given as relative roughness module: spherical NPs with smooth surface *w_PC,2_* = 1 while *w_PC,2_* is set to 10 for irregularly shaped NPs with sharp edges and ridges and a rough surface.

This is shown in Figure 2 for a NP with smooth surface *versus* a NP with a 10-times increased roughness.

3. Proposal for the third weighting factor *w_PC,3_*: The *zeta potential* (ZP) is the net electric potential formed by the charged groups of molecules of the NP surface and the surrounding medium. The ZP depends strongly on the pH of the medium: Commonly, absorption of proteins onto the NP surface may shift the ZP towards neutral ZP. For a quantitative estimate of ZP we use data recently published [32,33] and plot the relative neutrophil influx (NNI) in rat lungs *versus* the ZP (mV) of NPs of different materials which had been instilled 24 hours prior to broncho-alveolar lavage and subsequent NNI and ZP measurements.

**Figure 3:**
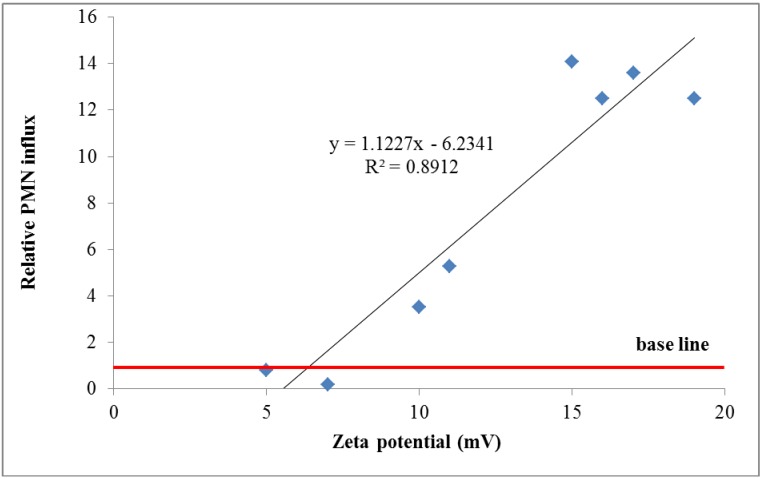
Weighting factor 3 (*w_PC,3_*) is depending on the zeta potential: The weighting factor is proportional to the relative PMN influx and increases according to equation 16. However, for a low zeta potential, it is not less than 1 [29,32].

The neutrophil influx is normalized to baseline values of neutrophil influx observed with non-reactive NP and without NP. The observed increase of NNI was fitted by a linear trend [32,33] (Figure 3):

NNI = 1.12 × ZP − 6.23 for ZP > 6 mV

Therefore, we propose for the zeta potential ZP a weighting factor w*_PC,3_* (arbitrary value):

*w_PC,3_* = max{1, 1.12 × ZP − 6.23} (16)

**Figure 4:**
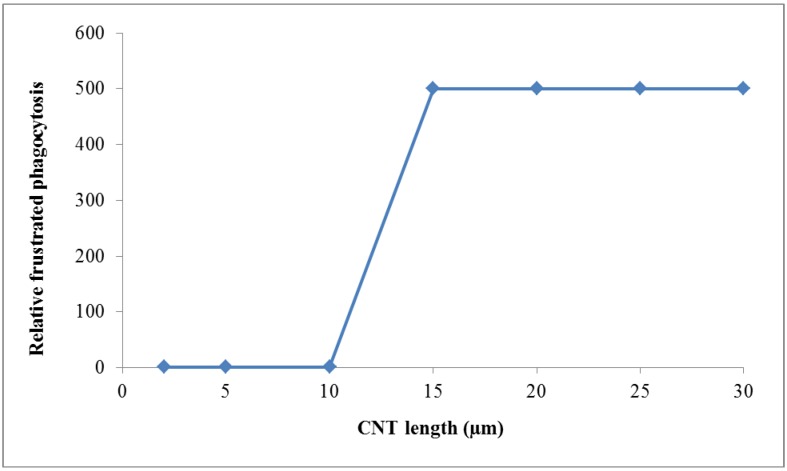
Weighting factor 4 (*w_PC,4_*) for particle morphology is proportional to frustrated phagocytosis of intratracheally instilled biopersistent and long carbon nanotubes (CNT): for normal phagocytosis *w_PC,4_* is set to 1 while the *w_PC,4_* is set to 500 for enhanced frustrated phagocytosis of CNT longer than 15 μm. We consider the risk of cancer induction and the risk of subsequent death as a most threatening event such that we chose a factor of 500.

4. Proposal for the fourth weighting factor *w_PC,4_*: The *w_PC,4_* is a weighting factor for the *particle morphology.* NPs may not only be spherically shaped, but elongated with a very high aspect ratio (ratio of NP length to NP diameter) like biopersistent and long carbon nanotubes (CNTs). We gather from the existing literature on CNTs and biopersistent asbestos fibers that the induction of free radicals and hence oxidative stress is caused by frustrated phagocytosis of lung macrophages ([33] and others) which cannot completely phagocytize CNTs with a length of more than 15 µm. This phenomenon has been shown in the literature and represents therefore a clear connection to the development of mesothelioma and subsequent death as shown by asbestos workers in the past. As an example we set normal phagocytosis without enhanced induction of ROS to unity and compare this to a high factor of 500 for enhanced frustrated phagocytosis associated with massive ROS induction and the potential for carcinogenicity. Due to the carcinogenic risk of developing mesothelioma we propose a high value for the weighting factor *w_PC,4_* (Figure 4):

*w_PC,4_* = 1 for NNI at normal phagocytosis *w_PC,4_* = 500 for NNI at frustrated phagocytosis (17)

**Figure 5:**
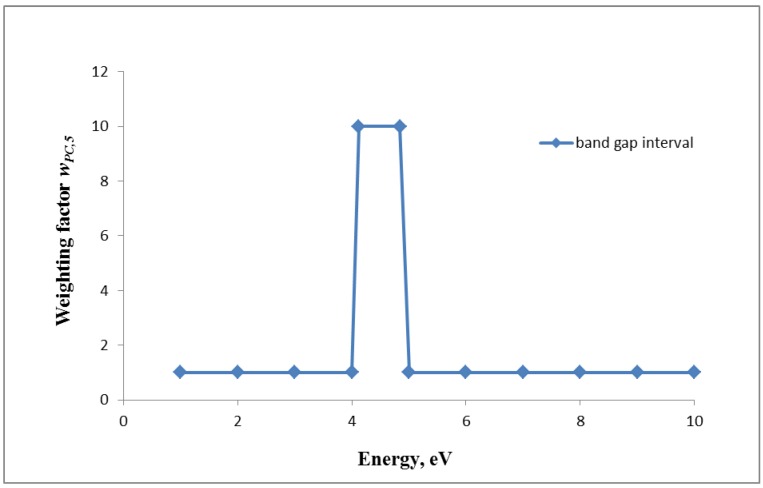
Weighting factor 5 (*w_PC,5_*) for the band gap energy levels of metal and metal oxides for NP larger than 20–30 nm: If NP is showing a band gap energy in the range of 4.1–4.8 eV, the *w_PC,5_* is set to 10 otherwise it is 1.

5. Proposal for the fifth weighting factor *w_PC,5_*: Burello and Worth [9] developed a theoretical predictive model for oxidative stress caused by metal and metal oxide NPs, based on the relationship between the cellular redox potential to *band gap energy levels* of metal and metal oxides. The authors suggest that these NPs with a diameter larger than 20–30 nm, whose band gap energy (E_c_) falls within the range of cellular redox potentials (−4.12 to −4.84 eV), are able to cause oxidative stress in biological systems. In a comprehensive study including *in vitro*, *in vivo* and multiparametric high throughput screening, Zhang *et al.* [34] confirmed this theoretical model for several metal and metal oxide NP materials. Therefore we propose a weighting factor for band gap energy levels (*w_PC,5_*). There are only very limited data which suggest that only in the selected band gap interval ROS formation occurs to a significant extent. From data cited in the literature we extrapolated a factor of 10 of the increase of weighting factor 5 as a plausible example. For NPs showing a band gap energy in the range of 4.1–4.8 eV we set *w_PC,5_* = 10, otherwise it is set to *w_PC,5_* = 1 (Figure 5):

*w_PC,5_* = 10 for NP atoms/molecules within band gap interval of 4.1–4.8 eV *w_PC,5_* = 1 for NP atoms/molecules outside the above band gap interval (18)

6. Proposal for the sixth weighting factor *w_PC,6_*: *w_PC,6_* is proposed to be a function of the *dissolution/dissociation rate* of NPd which has been shown to be proportional to the specific surface area SSA of most NP [35,36,37]. In fact, SSA is inversely proportional to the NP diameter d (Equation (1)) and proportional to the dissolution rate constant *k*; the latter is NP-material dependent and also depending on the physico-chemical properties of the cytosolic or body-fluidic solvents. According to earlier reports [38] the fractional loss of NP mass *m_NP_* over time *t* is:

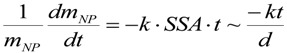
(19)
and the weighting factor *w_PC,6_* is proportional to the inverse of the diameter, the dissolution rate constant of the NP in the biological fluid and time *t*:

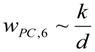
(20)

**Figure 6:**
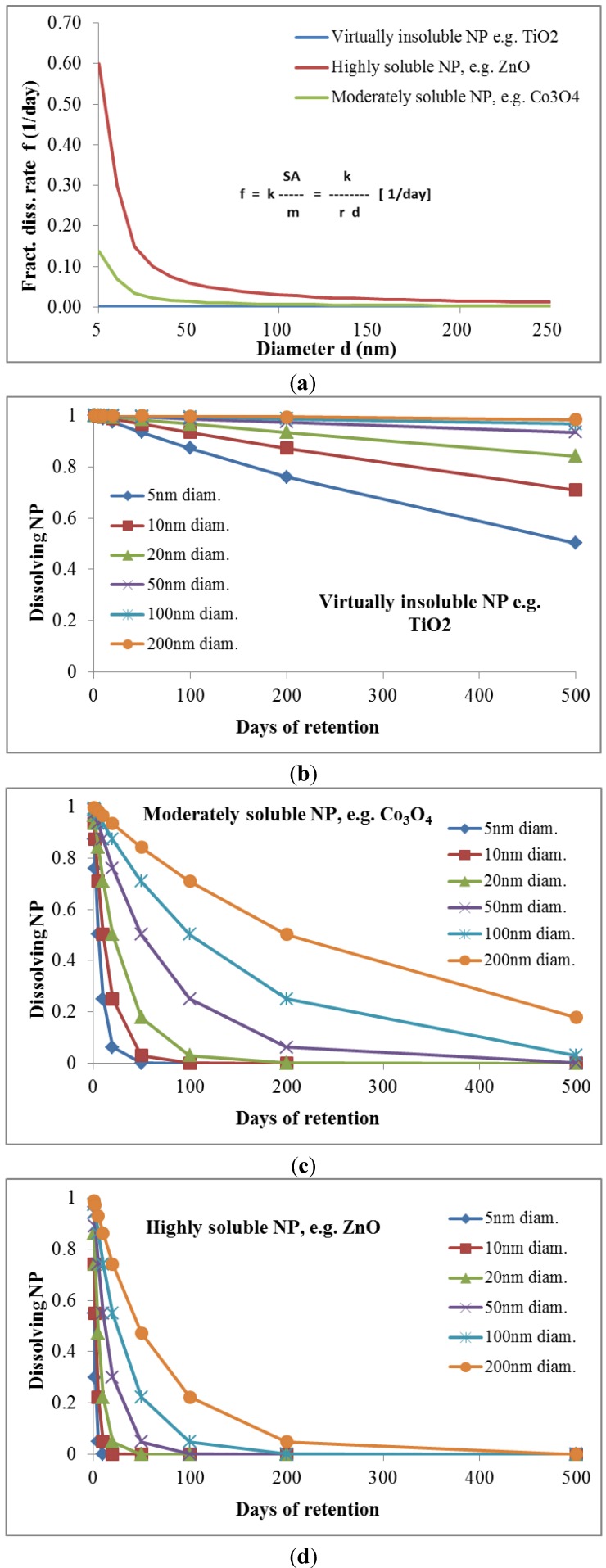
Weighting factor 6 (*w_PC,6_*) is a function of the dissolution rate of the NP. Panel **a**: fractional mass loss of differently soluble NPs over NP diameters ranging from 5 to 250 nm; Panels **b**–**d** give the fractional NP mass losses over time for different NP sizes of virtually insoluble (b), moderately soluble (c) and highly soluble NPs (d).

Changes of the fractional rate over time are shown in Figure 6 for NPs of different sizes and different dissolution rate constants. 

#### 3.2.3. Consequences for Equivalent Dose of NP

To summarize the above mentioned model, the equivalent dose of NP (*H_NP,T_*) is calculated by multiplying the deposited dose to the organ or tissue (*D_NP,T_*) with the nanomaterial specific weighting factors *w_PC,i_*. These factors consider the physico-chemical properties of specific NPs. The product of weighting factors *w_PC,i_* is proportional to the observed biological effect, of organ or tissue *T* for the same deposited NP dose. For weighting factors *w_PC,1_*, *w_PC,3_*, *w_PC,5_*, and *w_PC,6_* we used the equations provided in section “Equivalent dose of NP and weighting factors” and the corresponding figures. Due to the lack of *in vivo* and *in vitro* data regarding *w_PC,2_* and *w_PC,4_* we have suggested sets of data and provided their plausibility in section “Equivalent dose of NP and weighting factors”. These data were used to demonstrate how the deposited dose can be influenced by the physico-chemical properties of NP leading to the equivalent dose (*H_NP,T_*). 

**Figure 7:**
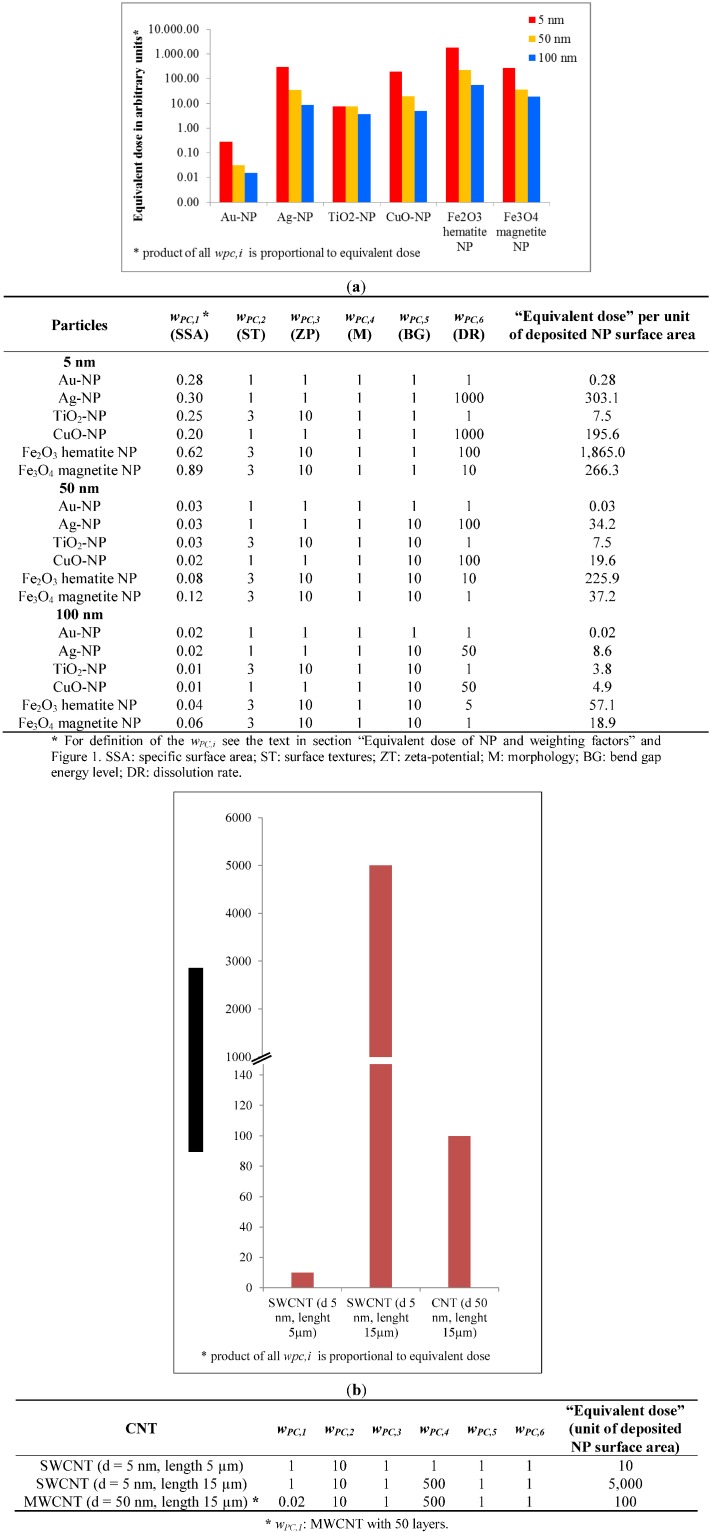
Comparison of the “equivalent dose” described by the product of all *w_PC,i_* for different NPs. The tables specify the *w_PC__,__i_* for each NP of 5, 50 and 100 nm diameter: (**a**) different metal and metal oxide NPs, and (**b**) for SWCNTs and MWCNTs. Note that in (**b**) Fe-ions of the dissolving Fe-oxide NPs play a key role in the Fenton reaction seen as a main source of ROS induction in biological systems, leading to oxidative stress. The tables indicate the calculation of the “equivalent dose”. For *w_PC,3_* we either presumed a zeta potential below 6 mV leading to a *w_PC,3_* value of 1 or a zeta potential of >15 mV leading to a *w_PC,3_* value set to 10 in the absence of detailed information.

Figure 7 shows the comparison of equivalent doses of different metal and metal oxide NPs being proportional to the product of the various *w_PC,i_*. For example 5 nm Ag-NP has a substantially higher *H_NP,T_* than TiO_2_ because the *w_PC,6_* of Ag-NP is 1,000-fold higher according to its very high dissolution rate constant and its very small diameter. CuO and Ag-NP are both rather soluble and the released ions were shown to induce oxidative stress or are bactericidal, respectively. Therefore, the product of the *w_PC,i_*—which is proportional to the equivalent dose—of both CuO and Ag-NP is determined by their high dissolution rate constants. It is interesting to see the differences of the *H_NP,T_* of hematite and magnetite form of Fe_2_O_3_. Again, the product of the *w_PC,i_* is determined by their different dissolution rate constants and sizes. As shown in Figure 7a, the equivalent dose for Au-NP is lowest since the values of *w_PC,2–6_* are minimal, while other NP—depending on their physico-chemical properties—show higher values for one of *w_PC,i_*.

In case of SWCNTs, the ratio of the surface atoms to the total = 1 since all atoms are on the surface. The *w_PC,2_* namely the surface texture is set to 10 since it is known that the form of the CNT and the length are relevant for the induction of biological effects. Moreover, it is known that the length-to-width ratio (aspect ratio) of the CNT is related to the “frustrated phagocytosis”. Therefore *w_PC,4_* is the main weighting factor which is set to 500 for ≥15 µm SWCNTs (Figure 7b), since we consider the development of mesothelioma including subsequent death (as has been proven by many asbestos workers) as the highest risk. For MWCNTs the *w_PC,1_* is different, depending of the number of layers and the correspondingly smaller ratio of surface atoms to total atoms, therefore the magnitude of the equivalent dose is 50-fold lower in this case. In summary, the application of *w_PC,i_* allows the comparison of the product of all weighting factors which is proportional to the equivalent dose of specific NP, and allows also a quantitative ranking or categorization of specific NPs. Figure 7 provides a first attempt how the equivalent NP dose can be ranked and it shows the huge differences between the various NP due to different physico-chemical properties. 

#### 3.2.4. Effective Dose

Only a few studies have investigated the sensitivity of organs, tissues and cells to NP-exposure (for review see [39]. In a recent *in vitro* study Sohaebuddin *et al.* [40] have shown that different cell types react with different sensitivity to the same NP exposure by expressing cell specific responses. However there are not enough adequate studies performed yet, to rank the sensitivity of organs, tissues or cells, whereby the sensitivity has to be compared with the same NP-dose under the same conditions. If the deposited dose and the equivalent dose are known, cell or tissue weighting factors might be introduced as was done for dose calculation of ionizing radiation. The relative biological effectiveness (RBE) or the biologically effective dose (BED; e.g., [20] can then be calculated as a function of the quality of the NP-exposure and the tissue/organ/cell. It has to be pointed out, that for radiation, tissue weighting factors are based on the concept of stochastic no-threshold radiation effects by linear extrapolation of available data of high radiation doses. For nanomaterials no data are available neither for long term exposure nor about sensitivity of organs and tissues. Therefore at present we cannot propose any tissue weighting factors, but it is suggested to identify sensitivities of organs, tissues, and cells, and then to apply it in analogy to radiation dosimetry:

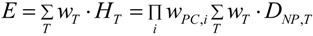
(21)

## 4. Conclusions

In the present paper we introduce a model for dose comparisons in analogy to the ionizing radiation dose model. The NP dose model includes the *deposited dose* (*D*) as the total deposited NP surface area per tissue mass in analogy to the absorbed radiation energy in biological tissue, the *dose rate* (uptake over time) and the biokinetics. The *equivalent dose* (*H*) takes into account the physico-chemical properties of different NPs. These physico-chemical properties are currently quantifiable and are included in the concept as NP-specific weighting factors. Furthermore, at present there are not enough data available to estimate the *effective dose* (*E*, weighted by the sensitivity of the biological material; tissue, organ, and cell), however we suggest to use specific organ/tissue/cell weighting factors when data will be available. The calculated equivalent doses for the different NPs in this paper show relative differences. Their actual values have to be determined in dedicated experimental investigations such as high throughput (HTP) in silico and *in vitro* assays. To progress systematically on the determination of weighting factors other relevant biological endpoint(s) have to be considered and tested. As it is known for ionizing radiation, data from a battery of endpoints indicate the biological effectiveness of the damaging agent. Similar approaches, by using modern HTP techniques, will allow identification of absolute numbers for weighting factors and differences on NP-equivalent doses. 

In summary, experience from radiobiology in generating a dose concept for deposited NPs seems to be very valuable. Thus, the implementation of deposited, equivalent and effective doses as well as dose rate according to the established radiation dose model could initiate further research and facilitate the understanding of NP-dose and related effects. The implementation of weighting factors based on different physico-chemical properties of specific NPs is an initial and useful tool to compare their equivalent doses. The presented NP dose model is meant to be a start for a NP dose assessment which needs further development. 
